# New Surgical Principles

**Published:** 1861

**Authors:** 


### NEW SURGICAL PRINCIPLES.

Prof. E. S. Cooper advances and urges discussion of the following surgical principles :

1st. That atmosphere, admitted into the joints or other tissues, is not a source of irritation or injury, except where it acts mechanically ; as, when admitted into a vein, by producing asphyxia ; into the thoracic cavity, by its pressure producing collapsing of the lungs, or when, by the long-continued exposure of a large amount of surface of any of the internal organs, whose normal temperature is much above that of the atmosphere, it reduces it so as to produce a morbid action.

2d. That the division of entire ligaments about the joints is no impediment to their ultimate strength and mobility ; but, on the other hand, this operation will often greatly facilitate the cure, by enabling the surgeon to open the affected part fully, for the purpose of applying medicinal substances to the articular surfaces, when these are ulcerated or otherwise diseased.

3d. That the only true mode of treating ulcerations of bone, however slight, within the joint, is to lay it open freely, and apply remedial agents directly to the part affected.

4th. That opening the joints early, in case of matter burrowing in them, is far more imperiously demanded than the opening of other parts thus affected, and the operation produces no further pain or inconvenience to the patient, in any respect, than when performed on parts remote from the joints.

5th. That after opening a large joint, the knee, for instance, by an incision several inches long, the wound should be kept open by the introduction of lint, or other similar substance, until the parts within the articulation become healthy, and, in all cases, it should be made to heal by granulation.

6th. That extensive wounds, opening freely the large joints, such as the knee, (even when lacerated, as by a saw, which must necessarily heal by granulation,) do not as often give rise to violent symptoms as very small wounds, such as are made by the corner of a hatchet, an adze, or a pen-knife, which heal on the outside by first intention.

7th. That there are no known limits beyond which a tendon will not or cannot be reproduced after division, provided the parts are made to heal by granulation, and that the present acknowledged rule of two inches being the maximum distance in which the divided ends of a ligament or tendon can safely be separated, has not the least foundation in fact.

